# The Symphony of Consumer Partnering and Clinical Governance: An Organizational Review Using the RE‐AIM Framework

**DOI:** 10.1111/hex.70095

**Published:** 2024-11-13

**Authors:** Jodie Nixon, Emily Steel, Warren Stubbs, Amber Williamson, Javed Khan, Phillip Carswell, Anne Coccetti

**Affiliations:** ^1^ Metro South Health, Clinical Governance, Risk and Legal Brisbane Queensland Australia; ^2^ School of Health and Rehabilitation Sciences University of Queensland Brisbane Queensland Australia; ^3^ Metro South Health, Clinical Governance, Risk and Legal (Consumer Partner) Brisbane Queensland Australia; ^4^ Metro South Health, Executive Services Brisbane Queensland Australia

**Keywords:** barriers, Clinical Governance, consumer partner, enablers

## Abstract

**Introduction:**

Partnering with Consumers in healthcare systems is now widely accepted and mandated in many countries. Despite this acceptance, there is minimal information regarding the best practice of how to successfully establish systems to embed this practice into healthcare systems.

**Methods:**

This evaluation used the RE‐AIM implementation framework to retrospectively analyse data from a 3‐year timeline to review the events relating to the transition of Consumer Partnering into a Clinical Governance Unit. Data was sourced via Phase 1 – a focus group to establish a 3‐year timeline of events, enablers and barriers, and Phase 2 – a quantitative and qualitative semi‐structured interview to review systems that had been developed to support embedding partnering with consumers into Clinical Governance.

**Results:**

Five primary enablers and five barriers to successfully embedding a Consumer Partnering Team into a Clinical Governance Unit were identified. Enablers included Executive sponsorship and ownership of the value of partnering with consumers, Executive leadership influence on local area uptake, an organization‐wide network, valuing via remuneration, and a centralized orientation and onboarding programme for Consumer Partners. Barriers included skills and attitudes of committee chairs, the size of the Directorate (smaller local areas can be easier to influence change), patient feedback data requires interpretation to be useful, staff turnover can reduce the relationships with Consumer Partners, and financial insecurity is a barrier to implementation and maintenance.

**Conclusions:**

This article described how an Australian Health Service embedded a Consumer Partnering Team into a Clinical Governance Unit to ensure that partnering became business as usual practice. Enablers, barriers, and unintended consequences can be used as learnings for other organizations to develop a similar approach.

**Patient or Public Contribution:**

Two Consumer Partners with lived experience of the health service, and members of the organizations committee structures are part of the evaluation team. As team members, the consumers participated as equal contributors in evaluation design, analysis of the focus group and interview data, and contribution to the writing and review of the manuscript. Two Consumer Partners with lived experience of the health service, and members of the committee structures participated in the focus groups and the interviews.

## 1 Introduction

Clinical Governance is the framework that health organizations use to ensure safe and quality care is delivered to every person who seeks support for health conditions [1]. In Australia, there are eight National Safety and Quality Health Service (NSQHS) standards that establish the minimum standard of care that a consumer can expect to receive from their local health service [2]. One of the eight standards is the Partnering with Consumer standard which mandates organizations to partner with consumers in their own care, and in the development and design of the organization [2]. Evidence demonstrating consumer involvement in strategy, policy, risk management, and workforce planning discussions and decisions is required, but how this is done is left to the discretion of each organization.

Embedding Consumer Partners into the Clinical Governance process of health services is considered effective and widely advocated for organizations [2, 3]. In 2023, a co‐produced qualitative evidence synthesis of 33 international studies established that successful formal partnering improves the person‐centeredness of health service culture and improves the physical environment of the health service [4]. A systematic scoping review identified that consumer engagement is required to continue to improve healthcare services but found insufficient research to guide the process [5]. It differentiated between engagement via patient advisory councils addressing day‐to‐day clinical challenges, versus patient members of governing boards deliberating on higher‐level operational matters [5]. A survey of Australian health services concluded that more easily organized engagement such as consumers on existing committees or reviewing patient resources are still the most widely used form of consumer partnering [6]. While less disruptive than changing existing structures, this type of engagement tends to run in parallel or ‘bolt‐on’ to health services rather than infiltrate the systems and involve consumers in strategic planning and activities considered more likely to affect population health [6].

To apply best practice principles [2, 4] and ensure that consumer partnering is not an afterthought [3], a roadmap for embedding consumer partnering in a health service governance is required. There are many challenges to changing practice in health services, including the gap between knowledge and its implementation, particularly in settings that are not optimal [7]. The RE‐AIM framework was developed to support the implementation or evaluation of programmes in a real‐world clinical setting [8, 9]. The five dimensions of the RE‐AIM framework each contribute a better understanding of retrospectively why an intervention did or did not work [8, 9]. The RE‐AIM framework evaluates, Reach – how much of the organization is willing to participate in the intervention; Effectiveness – impact the intervention is having on the organization; Adoption – organizational adoption of the intervention; Implementation – extent the intervention has been implemented across the organization, and Maintenance – extent the intervention has become a routine part of the organizations' practices [8, 9, 10]. The RE‐AIM framework was chosen to evaluate the transition of a Consumer Partner Team into a Clinical Governance Unit and to analyse the sustainability of consumer partnering for the organization moving into the future.

This paper aims to

1. describe and evaluate the process of embedding a Consumer Partner Team into a Clinical Governance Unit in an Australian health service; and

2. identify the successes and challenges that may affect the sustainability of consumer partnering in a Clinical Governance Unit to enable translation to other health settings.

## 2 Methods

### 2.1 Setting

The evaluation occurred at a public health service that includes six Directorates (local facilities) made up of five hospitals, Community and Oral Health, and Addiction and Mental Health Services [11]. The services provide specialist care to a population of more than 1.2 million people and employ more than 14,000 full‐time equivalent staff [11]. Exemption from ethical review was provided by the local Ethics Committee.

### 2.2 Design

The RE‐AIM framework was used to describe and evaluate the implementation of consumer partnering initiatives. The five dimensions (reach, effectiveness, adoption, implementation and maintenance) organized quantitative and qualitative data to identify real‐time implementation barriers and understand the how and why of implementation to enable translation in complex settings [8, 9].

Phase 1 – Focus group. Questions were developed by the first and second authors based on RE‐AIM framework to support the understanding of study Objective 1.

Phase 2 – Semi‐structured interviews. Questions were developed by the first author (JN) based on another study that utilized the RE‐AIM framework to organize quantitative data [10] to support the understanding of Objective 2.

The Standards for Reporting Implementation Studies (StaRI) checklist was used to ensure all important areas of implementation were considered in the evaluation [12].

### 2.3 Participants

Participation was voluntary and activities were conducted with consideration and organizational oversight to avoid their exposure to any harm. Remuneration was not available for participants. Five participants (including two of the Consumer Partners) were part of the authorship/evaluation team and took part in Phase 1 and Phase 2.

Phase 1 – Purposive sampling was used to recruit participants who were actively involved in consumer partnering at an organizational level from 2019 to 2022, when the Consumer Partnering Team moved from the organization's Strategy and Planning Team to the Clinical Governance Unit. The aim of recruitment was to get in‐depth knowledge of experience not diversity of perspective. The participants were selected as they were information‐rich sources on the topic [13]. Purposive sampling was used as it is widely used in implementation research that contains qualitative data collection. The method used was direct invitation from the first author (J.N.). Participants represented the Executive leadership team (four), Consumer Partners (two), and staff from the Consumer Partnering Team (two).

Phase 2 – Phase 2 included the same participants as Phase 1 with the addition of two staff from Directorate (facility) positions and one change with one of the Consumer Partners unavailable for Phase 2.

### 2.4 Data Collection

Phase 1 – A focus group was held in December 2022 with six participants to map a 3‐year retrospective timeline of events moving the Consumer Partnering Team into the Clinical Governance Unit. It was facilitated by another staff member who was not involved in the evaluation and supported by the first author (J.N.). The focus group explored enablers, barriers and unintended consequences of the transition. Two participants who were unable to attend the focus group were given the draft timeline and asked to contribute extra input, and then two focus group participants reviewed the draft and made additional observations.

Phase 2 – Participants were invited to complete an interview via an online session held between July 2023 and September 2023. The semi‐structured interview was conducted by the first author (J.N.) using the RE‐AIM framework to provide context for the quantitative data and the focus group results. The participants were given the interview questions before the interview to support reflective practice. The interviews focused on evaluating the systems implemented to support the consumers and staff working with the new Organizational structure and consumer feedback systems. Two of the ten participants being interviewed were directly line‐managed by the interviewer which may have contributed to relationship asymmetry. All participants were given the opportunity to review the final themes and provide feedback to an evaluation team member who was independent of the Consumer Partnering Team.

### 2.5 Data Analysis

Phase 1 – The first author (J.N.) transcribed the mapped data onto a timeline document that was emailed to all participants for verification and further input. Consensus was obtained via iterative email communication and document refinement until endorsed by all participants in August 2023.

Phase 2 – All semi‐structured interviews were audio‐transcribed and mapped to the interview questions on an Excel spreadsheet. Thematic analysis was completed by the first author (J.N.) with a review by the second author (E.S.) who was not involved in the interviews or part of the Consumer Partnering Team. Braun and Clark's inductive approach [14] was followed over six steps to develop patterns: 1. familiarity with the data (via the interview process and transcribing); 2. generating codes (conducted by J.N.); 3. searching the themes (conducted by J.N. and E.S.); 4. reviewing the themes (all evaluation team members including Consumer Partners); 5. defining the themes (J.N., E.S.); and 6. producing the report (all evaluation team members including Consumer Partners).

## 3 Results

### 3.1 Timeline Mapping

**Figure 1:**
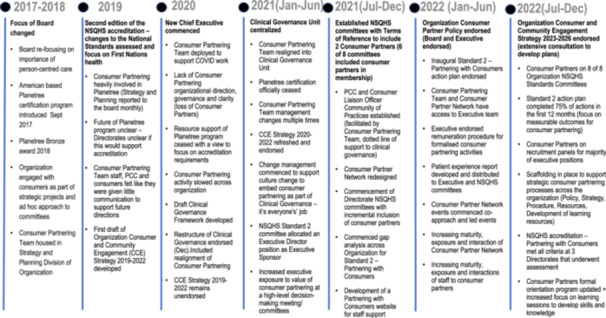
Timeline Mapping of transition of Consumer Partnering Team into Clinical Governance Unit. CCE, Consumer and Community; NSQHS, National Safety Quality Health Service; PCC, Person‐Centred Care Coordinators.

In 2017–2018, the Health Service Board introduced the American‐based Planetree‐certified programme [15]. The organization and Consumer Partners embraced the programme and committed to improving medical care with a person‐centred lens. In January 2019, the second edition of the NSQHS standards commenced, with person‐centred care requirements for accreditation detailed in Standard 2 Partnering with Consumers [2]. In 2021, the organization decided to cease Planetree certification and restructure Clinical Governance as a centralized department with a Consumer Partnering Team and include Consumer Partners as members on all NSQHS committees as part of a commitment to partnering. New committee structures were developed for each of the NSQHS standards, with Consumer Partners included as members. Several Consumer Partners left, and participants reported a lack of organizational direction for the Consumer Partnering Team during this period of change (Figure 1).

From July 2021 to December 2022, the organization invested significant development into the Partnering with Consumers' portfolio (Figure 1). This was led by an Executive Director sponsor and the NSQHS Partnering with Consumers committee (including four Consumer Partners) and supported by the Clinical Governance Unit and Consumer Partnering Team. Structures were established for the organization, including a Consumer Partnering Policy, linked procedures, a Consumer and Community Engagement Strategy and a Partnering with Consumers gap analysis and action plan to ensure everyone was working in a consistent direction. This was followed by further work to improve consumer input, including the formalization of a Consumer Partner Network (central orientation, quarterly meetings with executive and learning opportunities) and the development of a Patient Experience report containing data on complaints and compliments, patient stories and benchmarked (state‐wide) trended patient experience measures (Figure 1). Embedding Consumer Partners within structural governance, with clear strategic direction, coupled with strengthening patient experience and consumer support, allowed the transition from reactive partnering to proactive partnering.

### 3.2 Themes – Organizational Systems to Support Partnering With Consumers

The semi‐structured interviews evaluated the systems established to support Partnering with Consumers at an organizational level. This focused on policy documents, structures such as staffing and committees, and consumer feedback systems. The current consumer feedback systems used include Patient Reported Experience Measures Surveys (PREMS) as part of a state‐wide programme that reports real‐time patient experience data, Best Practice Australia (BPA) annual surveys conducted as a patient satisfaction review, and the complaints and compliments system. Table 1, 2, 3, 4, 5 summarizes the quantitative and qualitative RE‐AIM dimensions. The qualitative reflections were analysed separately from the perspectives of Executives, Consumer Partners, Consumer Partner Team members and Directorate representatives.

**Table 1:** Reach – Organizational systems to support Partnering with Consumers.

Reach – Quantitative						
Question			Measure			
How many Directorates are participating in the intervention (Partnering with Consumers as an Organization)?			Organization level + 6/6 Directorates			
How many Directorates have a Standard 2 committee (including CP members)?			6/6 Directorates			
How many Directorates have a dedicated role supporting Consumer Partnering?			4/6 Directorates			
How many Directorates/MSH have Consumer Partners linked to their services?			Organization level + 6/6 Directorates			
How many Organization NSQHS committees have CPs on the committee?			December 2022, 8/8 December 2019, 0/8			
How representative are the consumers who are involved?			December 2021 (*n* = 80) Gender Female 53% Male 36% Not stated 11% First Nations 9% Language other than English 25% Disability 31%		July 2023 (*n* = 111) Gender Female 63% Male 36% Nonbinary 1% First Nations 14% Language other than English 20% Disability 30%	
How many Directorates use consumer feedback (PREMS, BPA, Complaints) to inform gap analyses and action plans?			Organization level + 6/6 Directorates			

Reach – Qualitative Themes						
Question	Executive positions	Consumer Partners		Consumer Partner Team members		Directorate representatives
1. What were the barriers to centralizing the Consumer Partnering Team into Clinical Governance?	No barriers for two main reasons: (1) the changes made sense to people and were even seen as advantageous, so people supported and (2) earlier changes were perceived as more challenging than this process	Few barriers – rationale made sense and was supported but implications of change not always clear		Few barriers – accepted the direction and reason for the change but the transition took time and brought uncertainty for affected staff		Perceived as a threat by some and an opportunity by others. The structure was clear but the process and implications of change not easy to grasp
2. What were the barriers or variations for the Directorates to support the process of centralizing Consumer Partnering?	Historical issues of resource inequity surfaced. Change was not significant but strengthened leadership and relationships for better clinical governance	Seen as an external imposition by some with established systems, but others more enthusiastic about change		Systems already established, so change initially seen as threat to autonomy and brought uncertainty, but discussion brought support		Proposed structure made sense and not seen as big change given the historical trajectory
3. What were the barriers or variations for the MSH committees to support Consumer Partners onto Committees?	Uncertainty about new roles and relationships and getting it right. Practical and resourcing implications (remuneration, recruitment, orientation)	Change brought discomfort, inconsistency in leadership affected ease and success		Significant change to PwC team roles, mix of responses in committees – lots of work to establish committee structures and processes, upskill and support everyone for a unified approach		Feeling disconnected from and lacking confidence in the process
4. What were the barriers or variation for the Directorates to support Consumer Partners onto Committees?	Initial reticence of directorates – ability to find and train consumers, what value they might bring Lack of clarity of central versus site responsibilities to initiate and maintain	People had diverse experiences and weren't sharing or helping each other Skill set of consumers Getting the right fit for the consumer and the committee Each Directorate is very siloed		Good structure and support in place for the central team to directorate Some barriers and variations in responses but more welcoming than opposing		Needs leader and/or committee chair to value consumer partnering and support a change to status quo Committee chairs vary in how they perceive and perform roles (readiness and effectiveness) ‐ good partnerships are time‐intensive time constraints are barriers lack of dedicated PCC positions a barrier
5. Which committees/directorates still lack formal consumer partnering, and why?	Variation and evolution ‐ from understanding the value of partnering to doing it effectively in committees, easier in some settings than others	Clear differences between committees		Consumers not perceived as necessary on all committees, instead use primary (Standard 2) as mechanism for partnering		Improving with leadership and support Needs improvement
6. How representative are the consumers who are involved? (i.e., what is our target and reach in terms of diversity of consumers?)	Recognition of need for greater diversity, particularly youth Representation by numbers and dedicated committees	Representation via dedicated committees Improving representation by demographics Diversity of experience and nature of involvement of consumers matters – not numbers		Diversity has increased but could improve at local level		Difference between representing a population and having experienced the service Diverse consumers have been positive Consumers on committees don't represent the diversity of consumers in the hospital
7. What were or are the barriers or variation for Directorates to use consumer feedback (PREMS, BPA, Complaints)	Lots of data but limited analysis, and difficult to act Local action in response to feedback varies, pockets of good practice Room to improve	Working well – data gives an indication of what is happening and what people are doing Not working well – data is collected but not used or actioned		PREMS process is sub‐optimal for collecting and using data Investment required to seek feedback and act on it to make a real difference for consumers		Feedback is too general for clinicians to act on locally Focus is on quant rather than qualitative Investment required to investigate feedback/indicators for system improvement in partnership with consumersAbbreviations: BPA, Best Practice Australia survey; CP, Consumer Partner; PREMS, Patient‐ Reported Experience Measure Survey.

**Table 2:** Effectiveness – Organizational systems to support Partnering with Consumers.

Effectiveness – Quantitative					
Question			Measure		
Is there a current Organization Consumer and Community Engagement (CCE) Strategy? (Legislative requirement)			Yes		
What percentage of the CCE Strategy 2020–2022 was achieved?			The majority was achieved. The current CCE 2023–2026 strategy has commenced action on 71% of the 3‐year plan.		
Does the Organization NSQHS Standard 2 Committee have an action plan?			Yes		
What percentage of the Organization NSQHS Standard 2 action plan 2022 was achieved?			60% with the remaining rolled into the 2023 plan		
How many Directorates have a Standard 2 action plan?			4/6 Directorates		
How many sites underwent accreditation during this period (2019–2022)? Were there any Not Mets for Standard 2?			All sites. All met Std 2 requirements (One Directorate had 4 recommendations in late 2021, but met mid 2022)		

Effectiveness – Qualitative Themes					
Question	Executive positions	Consumer Partners		Consumer Partner Team members	Directorate representatives
1. What are the conditions and mechanisms that have led to the effectiveness of consumer partnering being embedded into Clinical Governance?	Good governance and management at organizational level from the MSH std 2 committee, PwC manager, and PwC team structure – executive sponsor, committee reps from each directorate, plans drive actions process – leadership, monitoring and communication from MSH to directorates	Direct contact between consumers and executives via meetings Change in committee strategy followed changed expectations of consumer partnering from national standards and board Central resourcing of clinical governance has helped directorates (with practical matters and building capacity)		MSH Partnering with Consumers Committee + action plan – clear vision and direction and everyone (staff and consumers) knowing what the focus is Aligned governance structure has improved effectiveness Alignment to organizational strategies	Accountability – plans are followed up teamwork – shared commitment from staff and consumers Support from PwC team – resources and point of contact
2. Have there been any downstream effects that were not expected?	Presence within clinical governance brings attention to PwC across teams and management (more so than other areas of organization, e.g., HEAT) with positive effect Consumer partners maturing faster than the organization Active involvement and support of executives makes advocacy for PwC easier at directorate level	Elevated importance of consumer partners Consumers contributions are taken seriously Redundancy and duplication of work at Directorates		Working with, rather than parallel to clinical governance Closer relationships with executive but further from the board Consumer partners and staff improving together with support Effective and better than expected	Visibility of Consumer Partnering/Team Increase workload at Directorates with new initiatives. Created a place for person‐centred Care Coordinators to network and collaborate
3. What explains variation on outcomes across sites?	Skill of committee chairs Directorate Culture – services linked closer to community tend to have more warmth, easier to build relationships in smaller sites Resourcing ‐‐ staff Size of directorate – big and complex structure is more difficult to influence	Staff changeover Commitment and passion of staff Some staff resistant to change Tension between central standardization and local tailoring		Directorates with different levels of maturity Consumers oriented to the service's structure and operations understand the issues	Size of directorate – bigger is more difficult Executive commitment
4. What are your thoughts on whether the staff/consumer partner relationship in committees is working as a partnership?	Discussion at meetings is collaborative Difference between partnership and advocacy or information sharing Variable and evolving – from well to tokenistic	I often feel that I'm part of the team Some instances it's still in developmental stage Incremental changes and improvements Negotiated how to work in genuine partnership Consumers can't approve/decide anything but can influence		There's positives and negatives. Some staff are really getting good at it and some staff are still not there yet Need to get good fit for partnerships to work	It's like any team as you get more used to each other and understand each other's styles and people get more confident Dependent on the chair Tough balance between dialogue (feels productive) versus bureaucracy (takes up valuable time)

Abbreviations: HEAT‐Health Equity Access Team, PwC‐Partnering with Consumers

**Table 3:** Adoption – Organizational systems to support Partnering with Consumers.

Adoption – Quantitative					
Question			Measure		
Has there been an increase in the number of Consumer Partners on Committees?			Organization NSQHS committees 8/8 have Consumer Partners 63 committees on the endorsed remuneration list. Nil formal remuneration pre‐2021, except for AMHS.		
Is there consistent use of consumer feedback (PREMS, BPA, Complaints/compliments)?			Quarterly patient experience report includes PREMS BPA well‐established Complaints and compliments reported at all Standard 1 committees and Board meetings		

Adoption – Qualitative Themes					
Question	Executive positions	Consumer Partners		Consumer Partner Team members	Directorate representatives
1. The restructure was led by multiple leaders. Which positions do you think made contributions and how?	External Clinical Governance Review – recommendation to move CPT into CG Chief Executive – made the decision ED Clinical Governance + Executive Sponsor + Director CG‐ strong interest Consumer Partnering Team – set the structure up to succeed	External Clinical Governance Review – recommendation to move CPT into CG Chief Executive + Board – made the decision ED Clinical Governance + Executive Sponsor + Director CG – strong interest Manager Consumer Partnering Team + team – set the structure up to succeed		ED Clinical Governance + Executive Sponsor + Director CG – strong interest Manager Consumer Partnering Team + team – set the structure up to succeed/strong vision Director Partnerships – handed over trust to CPT + consumers (not mentioned by consumers)	Unsure
2. What affects Organization/Directorates participation in PwC?	NSQHS Standards The business of healthcare can be overwhelming – consumer partnering can get overlooked Strength of relationships and influence of people can affect variation. Strong executives buy in, will increase focus on consumer partnering	Size of facility influences intimate contact Success breeds success		Executives buy in – Executives walk the walk Formation of Person‐Centred Care (PCC) Community of Practice brought directorates onto the same page	Executive Leadership commitment Experience and interest of Chairs Having PCC position Having systems to make partnering easier (remuneration, orientation)
3. Who is still not included (e.g., work areas, types of consumer reps) and why? Who have we/should we target for participation?	Unknown with infrastructure and planning Consultation at commencement of Strategic planning Nonclinical divisions – finances, disaster planning, corporate services	Clinicians (formal partnering) Always be non‐believers		Unknown with infrastructure and planning Formal partnering – still evolving in COH and AMHS	Nursing and medical buy in Corporate and admin services

Abbreviations: ADMS, Addiction and Mental Health; BPA, Best Practice Australia; CPT, Consumer Partner Team; ED, Executive Director; CG, Clinical Governance; COH, Community and Oral Health; PCC, Person‐Centred Care; PREMS, Patient‐Reported Experience Measure Survey.

**Table 4:** Implementation – Organizational systems to support Partnering with Consumers.

Implementation – Quantitative					
Question			Measure		
Was the Consumer Partnering Team embedded into Clinical Governance Unit as per the Clinical Governance Framework?			Yes		
Did the structural change of the Consumer Partnering Team into the central Clinical Governance Unit support the implementation?			Yes		
Implementation – Qualitative Themes					

Question	Executive positions	Consumer Partners		Consumer Partner Team members	Directorate representatives
1. What were (if any) the modifications to the intervention and why did they occur?	Implemented as intended as per Clinical Governance Framework	Leadership – genuine commitment		Handbook for Committees – consumer partner inclusion PCC Community of Practice Executive Sponsor – vision of how to implement Link to National Standards	Alignment to National Clinical Governance Framework
2. What were the barriers to the implementation as originally intended?	No barriers Recruitment of manager CPT Delay in streamline reporting structure for CPT	Some reluctance to centralize from Directorates due to loss of autonomy		Time to understand the change in work focus (Planetree to National Standards) Need more support at Directorates to help implement projects	Time to understand the change in work focus (Planetree to National Standards)
3. What were the contextual factors and processes underlying barriers to implementation and how do we address them?	Minimal barriers Some logistics‐ folder access	Improvements occurring have demonstrated the benefits of the change		Strength in developing things as a system versus one of project Strong manager vision	Building relationships to build trust and confidence Setting committees up for success (supporting chairs)
4. What is the process for which staff can improve their skills?	Having one stop shop for resources Having an expert team to support and grow others Online modules More coaching and mentoring Closing the loop and showcasing success Visibility of Consumer Partnering – what you see if what you accept	Face‐to‐face training workshops Executive leading by example Exposure of clinicians to successful consumer partnering Readiness of staff		Having one stop shop for resources Having an expert team to support and grow others Online modules More coaching and mentoring Not increasing clinician's workload Willingness of staff and priority against other competing demands	Support to committee chairs to understand consumer partnering Having an expert team to support and grow others More coaching and mentoring Closing the loop and showcasing success Learning together as staff and consumer partners

Abbreviaton: CPT, Consumer Partner Team

**Table 5:** Maintenance – Organizational systems to support Partnering with Consumers.

Maintenance – Quantitative					
Question			Measure		
Are there policy documents to guide partnering with consumers in the organization?			Consumer Partnering Policy Complaints feedback management procedure Using Australian Charter of Healthcare Rights procedure Remuneration and Reimbursement procedure Orientation, onboarding and exit procedure		
Is the Consumer Partnering Team maintained in the Clinical Governance structure?			Yes. 5.1 full‐time equivalent positions in the MSH team, 3.4 full‐time equivalent at 4/6 Directorates		

Maintenance – Qualitative Themes					
Question	Executive positions	Consumer Partners		Consumer Partner Team members	Directorate representatives
1. In what form are the components of PwC in Clinical Governance sustained?	Requirement of a central Consumer Partnering support team Requirement of Directorate staff to embed consumer partnering work Keep the consumer partnering conversation alive/importance of consumer partnering/show the difference we make/KPIs to demonstrate success Budget and FTE for consumer partnering support positions centrally and in Directorates Face‐to‐face meetings to build relationships	Face‐to‐face meetings Keep the consumer partnering conversation alive/importance of consumer partnering/show the difference/improvements we make/KPIs to demonstrate success value Budget and FTE for consumer partnering support positions centrally and in Directorates Feedback and recognition for consumers Succession planning		When the organization is aligned with focus and direction, miracles happen Systems thinking approach for sustainable programmes Consider direct line management of PCC positions to CPT to strengthen implementation	Central system to support policy documents Systems thinking approach for sustainable programmes Celebrate the wins to generate ongoing energy Need to strengthen the Directorates ability to implement and sustain programmes
2. What are the modifications that have been made at each Directorate after the transition of PwC into Clinical Governance?	Remuneration for consumer partners The PCC roles‐ need strong links to the Directorate Standard 2 committee	N/A		Uptake of implementation of centralized projects (reduces duplication) Support from the central Consumer Partnering Team	N/A
3. What are the barriers to maintaining the programme?	Staffing to support consumer partnering at the Directorates (knowledge + do the work) Provide an environment where consumer partners want to work with staff Financial constraints Executive support (if there is turnover) Managing performance of consumer partners	Matching consumer partners with committees/groups where they add value Staff turnover/staff shortages Being able to demonstrate genuine partnering for short‐notice accreditation Performance/effectiveness of consumer partners Managing performance of consumer partners Instability and frequent change within the organizations (constraining relationships) Competing organizational priorities and urgency		Staying current with best practice Having systems in place to support expectations of consumer partners Having the staff levels to support such a large organization to make sure implementation of initiatives is spread Need to make consumer partnering easy for staff to achieve Moving from implementation to business as usual	Budget and competing priorities Budget to support consumer involvement in quality improvement A person employed as a consumer partner
4. For Consumer Partners what is the succession plan, and intended rate of turnover?	Need to make sure we keep experienced people but also get fresh ideas Make sure we have grassroots service users as professional consumers can miss what's happening on the ground	Succession planning is difficult as people don't want to go Need to make sure we keep experienced people but also attach new consumers Staff also don't like to rock the boat (There needs to be) strategy for transition		Make sure there is a pathway for consumers to develop Review the effectiveness of the partnerships Matching consumer partners with committees/groups where they add value Need stronger links to community groups	Build skills in consumer partners and keep them Second staff to consumer partnering team to build skills Never have enough as there's always attrition

Abbreviatons: FTE, Full time equivalent; PCC, Person‐Centred Care; PwC, Partnering with Consumers.

#### 3.2.1 Reach – Organizational Systems to Support Partnering With Consumers

*Reach – How much of the organization is willing to participate in partnering with consumers* (Table 1)*?*

Partnering with Consumers is occurring at the organizational level and at all Directorates. This is evidenced by Consumer Partner representation on eight of the organization's NSQHS committees, and on all the Directorate Partnering with Consumers Committees. All Directorates are using consumer feedback systems, although it is difficult to establish the extent the information is influencing service improvement. Current Consumer Partner demographics include more females (63%) than males (36%), First Nations representation of 14% because of a focus to establish a First Nations Elders Advisory Group in 2022, 20% who speak a language other than English at home, and 30% who identify as living with a disability.

There were few barriers to centralizing the Consumer Partnering Team into Clinical Governance as the changes made sense to people and were seen as advantageous; however, the transition time and implications of change were unsettling for Consumer Partners, the Consumer Partner Team, and the Directorate. The Directorates gave mixed reports, with some feeling that change was imposed externally despite established systems, while others were more enthusiastic about the change. Some historical issues of resource inequity surfaced in Directorates, while Executives felt the change strengthened leadership and relationships for better clinical governance.

*“A cultural change happening and a mindset change, some more enthusiastic about it than others” (Consumer Partner)*.

*“Potential concerns about the unknown, like what it would mean working with consumers or not understanding what value that they might bring to the table” (Executive)*.

The introduction of Consumer Partners onto the NSQHS committees was a significant role change for the Consumer Partnering Team who were tasked with the recruitment, orientation, and onboarding of Consumer Partners; however, the centralization of this process improved the structure and support offered to the Directorates. There was uncertainty about the new roles and relationships and getting it right, which brought discomfort. Consumer Partners and Directorate staff emphasized that the executive leader and/or committee chair needs to value consumer partnering and support the change to status quo to develop good partnerships that lead to change.

*“So, I think some of the barriers also is of how consumer partnering is valued, embraced, accepted, promoted by the executive at the at the time and depending upon who's in those executive positions” (Directorate)*.

#### 3.2.2 Effectiveness – Organizational Systems to Support Partnering With Consumers

*Effectiveness – what impact is partnering with consumers having on the organization* (Table 2)*?*

The organization has a current Consumer and Community Engagement Strategy, which is a legislative requirement, and an NSQHS Partnering with Consumers action plan to implement the strategy and ensure the criteria of the Nationals Standards are met. Four of the six Directorates also have an action plan, and all met the requirements of the Partnering with Consumers standard during accreditation assessments conducted between 2019 and 2022. One of the Directorates assessed in 2021 was asked to address four recommendations to improve training of Consumer Partners and the workforce. The centralization of orientation, onboarding and learning supports have assisted in meeting these recommendations.

*“When consumer partners are part of the gap analysis and action planning, they can meaningfully engage and are able to understand what all is about” (Consumer Partner Team)*.

The alignment of the Consumer Partnering Team into the governance structure has improved effectiveness, enabled by a dedicated NSQHS Standard 2 committee that has an action plan with a clear vision and direction for staff and consumers. The committee structure has an executive sponsor and committee representative from each Directorate to drive actions. The Consumer Partnering Team, including the Manager position that chairs the committee, provides a point of contact and accountability to ensure that plans are followed and contribute to organizational strategy.

*“Coming from the top of the organization all the way through, with governance then providing the structures and frameworks for that (consumer partnering) to actually happen” (Executive)*.

Some downstream effects to the transition of Partnering with Consumers into the Clinical Governance Unit included the increase of visibility of the Consumer Partnering Team and the positive influence on other teams in the unit, the access to Executives, and the elevated importance of the contributions of Consumer Partners. Participants opinions varied as to whether the staff and Consumer Partners are working as a partnership, with the acknowledgement like any team, familiarity with each other's styles increases confidence in the relationship. There was a general sense among participants of incremental changes and improvement occurring with the building of the staff/Consumer Partner relationship, with variation mostly attributed to the committee chairs.

#### 3.2.3 Adoption – Organizational Systems to Support Partnering With Consumers

*Adoption – how much of the organization is partnering with consumers? How many people are actively involved* (Table 3)*?*

There has been widespread adoption of consumer partnering, not only on the NSQHS committees but also in advisory committees (First Nations Elders Advisory Council, Multi‐cultural and Disability and Inclusion), development of strategic plans, and staff recruitment processes. An active recruitment drive has increased the number of formal consumer partners from 80 in 2019, to more than 110 in 2022. Remuneration for formal partnering activities is offered to Consumer Partners on over 63 committees. Consumer feedback systems include PREMS, BPA, and complaints/compliments, reported to clinical, executive and Board meetings at regular intervals. Despite the organizational reporting, there is little oversight into how consumer feedback contributes to service change and improvements.

Factors contributing to the strong adoption across the organization include Executive leadership, support from the Manager of the Consumer Partnering Team to establish a structure, a clear vision that was inclusive of Consumer Partners and Directorates, and the systems such as orientation and remuneration to make partnering easier.

*“So, the hinge point was taking consumer partnering and bringing it within the framework of governance. This was a hinge point in the growth and development and support of consumer partnering” (Consumer Partner)*.

#### 3.2.4 Implementation – Organizational Systems to Support Partnering With Consumers

*Implementation – to what extent has partnering with consumers been implemented across the organization* (Table 4).

The Consumer Partnering Team has successfully transitioned into the Clinical Governance Unit as per the organization's Clinical Governance Framework, which is based on the National Clinical Governance Framework [1]. The structural change has enabled several support programmes to be developed and implemented including the NSQHS Partnering with Consumers Committee, the Person‐Centred Care Community of Practice, Consumer Partnering Orientation, Consumer Partner remuneration, and the Consumer Partner Network. These programmes are all supported by procedures and the Consumer Partnering Team.

*“Having those Person‐Centred Care Coordinators is such an important position to facilitate person‐centred care and the understanding for staff to embrace what that means to them, and how it actually should be just a part of their job that they communicate with their patients in that partnership model” (Directorate)*.

Use of a systems approach to develop and sustain the programmes is seen as an enabler of implementation. The structures have enabled relationships to develop between staff and consumers, which has built trust and confidence in progressing actions and improvements. Ongoing challenges to implementation across the organization include limited resources to support consumer partnering, and the time constraints placed on staff who have multiple competing demands. This has been reported as a challenge through all the RE‐AIM dimensions.

#### 3.2.5 Maintenance – Organizational Systems to Support Partnering With Consumers

*Maintenance – to what extent had partnering with consumers become part of routine organization practice* (Table 5).

The organization deliberately chose to have an Executive and Board‐endorsed Consumer Partnering Policy that describes the ethos of partnering. The policy is supported by procedural documents that set minimum standards of business conduct to maintain the commitment to Partnering with Consumers. The organization employs 5.1 full‐time equivalent positions dedicated to this portfolio, in addition to 3.4 dedicated positions across the Directorates. These dedicated positions support a local population of more than one million people, alongside a general commitment from staff to the ethos of partnering.

*“We need the central team to support a lot of the work that that goes on, and resources within the Directorates to implement the work” (Executive)*.

Continued growth and successful consumer partnering is dependent on retention of experienced people alongside continued recruitment to bring fresh ideas and represent the diversity of the community. A pathway of development for consumers and process to match Consumer Partners with committees/groups where they add value is seen as critical to maintenance and growth.

*“I think it's also just being realistic with the planning and making sure that not biting off too much” (Directorate)*.

*“Need a sustainable model for recruitment of consumer partners so you're managing/supporting your future replacement” (Consumer Partner)*.

*“When the organization is completely aligned with focus and direction, magic happens” (Consumer Partner Team)*.

### 3.3 Unintended Consequences

The mapping of the timeline and the interviews revealed some unintended consequences as a result of moving the Consumer Partnering portfolio into the Clinical Governance Unit. The majority of these were viewed as positive downstream effects.

#### 3.3.1 Executive Positions

The presence of Consumer Partnering within Clinical Governance increased visibility across other areas within Clinical Governance (such as the Patient Safety Team, the Clinical Governance and Compliance Teams and the memberships of the Executive Quality and Safety Committee) with a positive effect and an increase in uptake of using Consumer Partners in activities such as accreditation processes and development of procedures. Consumer Partners were potentially maturing faster in their ability than the organization's acceptance.

#### 3.3.2 Consumer Partners

The transition of Consumer Partnering into Clinical Governance elevated the importance of Consumer Partners, with consumer contributions being taken seriously. There was a redundancy and reduced duplication of work being undertaken at the Directorates as system change was led by the central team, but then implemented as appropriate at the Directorate.

#### 3.3.3 Consumer Partner Team

The transition of Consumer Partnering into Clinical Governance enabled a seamless working together model rather than working in parallel with clinical governance. It also enabled closer relationships with executive leadership, but potentially more distanced from the Board. The change enabled Consumer Partners and staff to learn and improve together. It was more effective to achieve outcomes and progress changes than expected.

#### 3.3.4 Directorate Positions

It increased the visibility of Consumer Partnering and the central team. It created a place for Person‐Centred Care Coordinators to network and collaborate. One downstream effect was an increase in workload for Directorates with the introduction of new initiatives. Although the initiatives were welcomed and seen as desirable, there was often no additional resources to support the increase in workload.

## 4 Discussion

Our evaluation used the RE‐AIM implementation framework to explore the enablers and barriers to embedding a Consumer Partnering Team into a Clinical Governance Unit. A Cochrane review published in 2021 reviewed five trials with more than 469 health service providers to determine that, the effects of consumers and health providers working in partnership is still unknown, however, recommended that pragmatic studies and qualitative research, such as this implementation evaluation were required to build the evidence base for working together in healthcare [16]. This evaluation has not found new concepts; however, it builds on the current evidence by describing enablers and barriers to implementation.

### 4.1 Enablers of Consumer Partnering and Clinical Governance

Five main factors were reported as enablers of successful implementation of consumers partnering in this evaluation, and they are consistent with current literature.

1. Executive sponsorship and ownership of partnering with consumers is essential, supported by strategic plans and action plans. National policy does not specify how to partner to ensure equity of power and success in consumer relationships [3]. Executive ownership and a clear vision in local policy documents support harmony in partnering [4, 5]. This organization has achieved this by allocating the Consumer Partner portfolio to an established Executive Director position, in addition to the Consumer Partner Team portfolio sitting in the Clinical Governance Division which is also governed by an Executive Director position. This ensures that Consumer Partnering is consistently advocated for at an executive level.
2. Executive leadership and services directly connected to communities influence local (Directorate) culture. Adoption of formal consumer partnering is easier when it builds on existing relationships within the community. This is consistent with the Merner et al. (2023) Cochrane review and the Sagen et al. (2023) systematic review. Additionally, as supported by Cox et al. [17], the leadership team has a key role in fostering, resources and promoting the connection between the organization and Consumer Partners. This connection can occur before and during committee meetings or during attendance at the Consumer Partner Network meetings. Executive need to be available to respond to questions and concerns via email, newsletters and in person meetings.
3. An organization‐wide network enhances visibility and strengthens relationships alongside a central Consumer Partnering Team and collaboration with local Person‐Centred Care Coordinators. This makes it easier to implement strategic directions and action plans into local facilities and is consistent with multiple other studies [4, 5, 18], This organization has achieved this by having a central Consumer Partner Team and directorate‐based Person‐Centred Care Coordinators that support the Consumer Partner Network, develops the quarterly newsletters, and encourages staff to close the loop on engagement outcomes and influences.
4. Valuing Consumer Partners via remuneration demonstrates the value placed on partnering. This is extensively supported in the literature as remuneration is considered essential to ensure that the diversity of the community is represented, by compensating people for their time [5, 19, 20, 21]. This organization initiated a central remuneration budget that is supported by a procedure to ensure consistent remuneration practices with Consumer Partner activity. Rates are linked to Health Consumers Queensland recommendations [22].
5. Centralized orientation and onboarding of Consumer Partners, supported by dedicated and local Person‐Centred Care Coordinators, improves, and sustains engagement of Consumer Partners. This is extensively supported in the literature [4, 5, 20]. This organization achieved this by establishing a central orientation programme that all new Consumer Partners who will be engaging with the organization in an ongoing formal capacity attend (with remuneration) which then links to the local work area to support onboarding to the local project, committee, or quality improvement project.

### 4.2 Barriers to the Symphony of Consumer Partnering and Clinical Governance

Five prominent barriers were identified by participants in the evaluation.

1. The skills and attitudes of committee chairs are significant in whether and how Consumer Partners are involved in committees and projects. Committee chairs who embraced Consumer Partners had significantly improved outcomes with interactions [4, 5, 17, 20]. It is recommended that a simple training or peer mentor programme be available to support committee chairs to understand effective partnering with consumers.
2. A barrier that this evaluation observed that has not previously been reported in consumer engagement literature is the size of Directorates/facilities makes a difference in change management with the bigger the facility associated with increased difficulties implementing change. Executive sponsorship and ownership can influence the culture change to support embedding partnering with consumers [17]. For example, a unique programme this organization initiated was the introduction of a Consumer Partner panel as part of the recruitment process for Executive positions which demonstrates the organization's value and intent for authentic partnering.
3. Large quantities of patient experience data cannot contribute to improvement without analysis. Feedback is often too general for clinicians to act on locally. This is consistent with other studies that have found patient experience data is collected; however, collation and thematic interrogation is not common [23]. Organizations need to develop a mechanism to analyse and use patient experience data to support the identification of gaps and to develop quality improvement directions.
4. A unique yet expected finding from this evaluation is the way that staff turnover can affect the continuity of connection between staff and Consumer Partners. The loss of experience and knowledge in relation to consumer partnering associated with staff turnover can also limit the speed or capacity to drive initiatives. Programmes that can continue to build connections such as a Consumer Partner Network, a centralized Consumer Partner Team and Directorate‐based Person‐Centred Care Coordinator positions can ensure continuity and retention. If a staff member leaves a position, it is powerful for the existing staff member to introduce the new staff member to the project/committee Consumer Partners to ensure continuity of relationships.
5. Another area that has had minimal discussion in the literature is the ability for an organization to financially sustain the practice of consumer partnering. A few studies have touched on limited funding dedicated to partnering from policy‐makers as a barrier to implementation and maintenance [5, 24, 25]. Health services have multiple demands competing with consumer partnering initiatives. Successful Partnering with Consumers requires financial investment in staff, remuneration for Consumer Partners, and funding of initiatives such as learning and development programmes and translation of resources into multiple languages. Allocated resources need to be established to support this work rather than relying on the good will of staff or services to absorb this workload within already busy portfolios.

### 4.3 Limitations

This evaluation has limitations. It is limited by the bias of the evaluation team and contributors in favour of the changes made and the current system. Three of the seven authors are employed to design and deliver the programme being evaluated and therefore have a vested interest in its success. Different perspectives may have emerged from a wider consultation process. The RE‐AIM framework was used to compare and balance the perspectives of different stakeholders in the evaluation process. This evaluation was completed in a large public Australian health service, so the discussion points may not readily transfer to other health settings.

This evaluation has demonstrated that embedding a system of Consumer Partnering into a Clinical Governance Unit is worth integrating into a healthcare setting. One gap that requires further investigation is the development of an evaluation tool to explore the effectiveness and outcomes obtained from the partnering process and the translation into health outcomes.

### 4.4 Implications for Practice

Successful Consumer Partnering requires committed leadership and management, underpinned by an organizational culture that understands and values consumers as partners in the governance of health services. A dedicated Consumer Partnering Team located within the organization's Clinical Governance Unit can ensure that orientation, onboarding, support and remuneration are provided for consumer partners, and support provided to staff. Genuine change management in large organizations takes time, there will be many challenges before the incremental rewards of changed practice are experienced.

## 5 Conclusion

There are many challenges to changing practice in health services, including a gap between knowledge and its implementation, particularly in settings that are not optimal.

This article described how an Australian Health Service embedded a Consumer Partnering Team into a Clinical Governance Unit to ensure that partnering became business as usual practice throughout the organization. Enablers, barriers and unintended consequences will be used to continue the development of this ethos throughout our organization and can be considered as learnings for other organizations to develop a similar approach.

## Author Contributions

**Jodie Nixon:** conceptualization, investigation, writing–original draft, methodology, validation, visualization, writing–review and editing, project administration, supervision, formal analysis, data curation. **Emily Steel:** conceptualization, investigation, writing–original draft, methodology, validation, writing–review and editing, formal analysis, data curation, supervision. **Warren Stubbs:** conceptualization, investigation, writing–review and editing, validation. **Amber Williamson:** conceptualization, investigation, writing–review and editing, validation. **Javed Khan:** conceptualization, investigation, writing–review and editing, validation. **Phillip Carswell:** conceptualization, investigation, validation, writing–review and editing. **Anne Coccetti:** conceptualization, investigation, validation, writing–review and editing.

## Ethics Statement

This study received an ethics exception from the Local Research Ethics Committee.

## Conflicts of Interest

The authors declare no conflicts of interest.

## Data Availability

The data that support the findings of this study are available from the corresponding author upon reasonable request.
